# Anterior insular cortex glutamate-glutamine (Glx) levels predict general psychopathology via heightened error sensitivity

**DOI:** 10.3389/fnins.2025.1592015

**Published:** 2025-07-16

**Authors:** Haeorum Park, Minchul Kim, Jaejoong Kim, Sunghwan Kim, Bumseok Jeong

**Affiliations:** ^1^Graduate School of Medical Science and Engineering, Korea Advanced Institute of Science and Technology (KAIST), Daejeon, Republic of Korea; ^2^Department of Radiology, Kangbuk Samsung Hospital, Seoul, Republic of Korea; ^3^Department of Psychiatry, University of Minnesota, Minneapolis, MN, United States; ^4^Department of Psychiatry, The Catholic University of Korea Yeouido St. Mary's Hospital, Seoul, Republic of Korea

**Keywords:** reinforcement learning, general psychopathology, functional MRS, glutamate, error sensitivity

## Abstract

**Introduction:**

The anterior insular cortex (AIC) integrates interoceptive, cognitive-emotional, and error-monitoring signals, and is consistently hyperactive in anxiety and depression. Converging evidence links elevated glutamate + glutamine (Glx) in fronto-insular regions to stress reactivity; however, it is unknown whether AIC Glx relates to a transdiagnostic general psychopathology factor (G-score) or to the tendency to overweight prediction errors during learning. We therefore combined functional MRS (fMRS) with reinforcement-learning modeling to test whether (i) baseline AIC Glx predicts the G-score derived from bifactor analysis of PHQ-9, GAD-7, and STAI-X1, and (ii) task-evoked Glx changes track individual differences in error sensitivity during gain- and loss-based learning.

**Methods:**

Fifty-six healthy adults (22 ± 2 yr, 16 women) completed the questionnaires and performed a two-armed bandit task (40 loss then 40 gain trials) while single-voxel semi-LASER spectra were acquired from AIC and medial prefrontal cortex (mPFC) at rest and during each block. Six Rescorla-Wagner variants were fitted to the choices; the best model (based on the lowest LOOIC) included error sensitivity, decision temperature, and value decay. Glx (CRLB < 20%) was quantified using LCModel and analyzed with repeated-measures ANOVA and Bonferroni-corrected correlations; mediation was assessed using Baron-Kenny steps (α = 0.05).

**Results:**

Baseline AIC Glx correlated with the G-score (*r* = 0.39, *p* = 0.004) and with error sensitivity for gains and losses (*r*≈0.41–0.44, *p* ≤ 0.005); mPFC Glx showed no such relations. AIC Glx fell during gain learning (−2.21%, *p* = 0.034) and remained low post-task, whereas mPFC Glx was unchanged. Error sensitivity fully mediated the AIC-Glx/G-score link; associations were specific to Glx, not other metabolites.

**Discussion:**

Higher excitatory tone in the AIC appears to enlarge prediction-error weighting, which in turn amplifies a shared anxiety-depression dimension. Dynamic Glx reductions during reward learning suggest acute metabolic demand superimposed on a trait-like baseline that biaes cognition. Targeting insular glutamatergic function–pharmacologically or via neuromodulation–may therefore mitigate maladaptive error processing that underlies internalizing psychopathology.

## 1 Introduction

The anterior insular cortex (AIC) is a pivotal brain region involved in a wide range of cognitive and emotional processes, including interoception (Dobrushina et al., 2020), error detection (Palermo et al., 2018), and risk assessment (Gogolla, 2017; Paulus and Stein, 2006). Its unique anatomical and functional connectivity positions the AIC as a critical hub for integrating sensory information with emotional states, making it particularly relevant in the context of anxiety and depression (Wager and Barrett, 2017). These mental health conditions are often characterized by heightened sensitivity to negative outcomes and an exaggerated response to perceived threats or errors, functions that are closely tied to AIC activity (Paulus and Stein, 2006; Wager and Barrett, 2017).

The involvement of the anterior insula in psychopathology, particularly in anxiety and depression, has been supported by numerous neuroimaging studies (Gogolla, 2017). Functional MRI (fMRI) research has consistently shown that the AIC is hyperactive in individuals with anxiety disorders during tasks that involve uncertainty or error monitoring (Paulus and Stein, 2006). For example, increased AIC activation has been observed in response to negative feedback during decision-making tasks, a response that correlates with the severity of anxiety symptoms (Paulus and Stein, 2006). Similarly, in depression, the AIC has been implicated in the processing of negative emotional stimuli, with increased insular activity being associated with rumination and negative affect (Wager and Barrett, 2017).

The concept of the general psychopathological factor score, or “G-score,” which captures the shared variance across various mental health symptoms, including anxiety and depression, has gained prominence in recent years (Caspi et al., 2014; Lahey et al., 2017). The G-score provides a more holistic understanding of psychopathology, recognizing the overlap between different mental health conditions. Previous research has suggested that this G-score is not merely a statistical artifact but may be underpinned by common neurobiological mechanisms (Lahey et al., 2017). Moreover, because anxiety and depression symptoms are highly comorbid and often show substantial overlap even in subclinical samples, focusing on a single general factor enhances statistical power and reliability compared to analyzing each scale separately. In healthy or non-clinical populations, individual PHQ-9 and GAD-7 scores frequently exhibit floor effects and restricted range, which can obscure true association with neurobiological measures. By applying bifactor modeling to large independent normative datasets, we derive a latent G-score that captures shared variance across multiple instruments while partitioning out domain-specific noise and reducing measurement error. This transdiagnostic approach aligns with emerging frameworks in psychiatric research, emphasizing common neurobiological substrates across internalizing disorders and enabling detection of Glx-psychopathology relationships that might be missed when anxiety and depression are examined in isolation.

Glutamate is the primary excitatory neurotransmitter in the brain, playing a central role in synaptic plasticity, learning, and memory (Marsman et al., 2014). Alterations in glutamatergic signaling have been implicated in various psychiatric conditions, including anxiety, depression, and schizophrenia (Moriguchi et al., 2019; Marsman et al., 2014; Sanacora et al., 2012). For instance, elevated glutamate levels in frontal and cingulate brain regions have been linked to increased stress responsiveness and anxiety, suggesting that hyperglutamatergic signaling in these regions may underlie heightened arousal and maladaptive stress reactions. Similarly, in major depressive disorder, dysregulated glutamate metabolism—characterized by regional alterations in glutamate and glutamine concentrations—appears to be a fundamental mechanism connecting glutamatergic neurotransmission changes to depressive symptomatology (Sanacora et al., 2012). Specifically, in the context of anxiety and depression, glutamate's role in modulating neural circuits involved in error processing and emotional regulation is of particular interest (Arnone et al., 2015; Nasir et al., 2020).

Functional magnetic resonance spectroscopy (fMRS) studies have provided valuable insights into the neurochemical landscape of these disorders, showing that glutamate levels can be altered in key brain regions like the AIC (Park et al., 2018). However, the relationship between glutamate concentrations, especially the Glx complex (a combined measure of glutamate and glutamine), and general psychopathology remains underexplored (Deelchand et al., 2018).

The role of the AIC in learning from errors and decision-making further underscores its relevance to mental health (Paulus and Stein, 2006). The AIC is activated during tasks that involve processing prediction errors—the difference between expected and actual outcomes—particularly in contexts that involve potential losses (Addicott et al., 2021). Error sensitivity is a critical component of adaptive learning, allowing individuals to update their beliefs and strategies based on feedback (Wagner and Rescorla, 1972). In the context of psychopathology, however, this process can become maladaptive. Individuals with anxiety or depression may overestimate the significance of errors or negative outcomes, leading to heightened stress and maladaptive decision-making (Aylward et al., 2019; Tobias and Ito, 2021).

Accordingly, our primary aim was to characterize how AIC Glx concentrations fluctuate across reinforcement learning—specifically during gain and loss blocks—and to test whether these task-related changes relate to individual differences in error sensitivity. Our secondary aim was to examine whether baseline (pre-task resting) AIC Glx levels are associated with a trandagnostic general psychopathology factor that captures shared variance in anxiety and depression symptoms.

**Figure 1:**
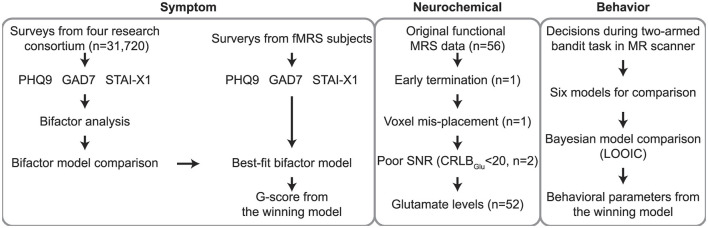
Overall workflow of the study. First, general psychopathology scores (G-scores) for anxiety and depression were calculated based on self-reported questionnaire data (PHQ-9, GAD-7, STAI-X1) obtained from participants (*n* = 31,720) of the Young Adulthood Depression Research Consortium (Choi et al., 2021), the parent cohort of this study. Utilizing the bifactor model, which best explained responses on depression and anxiety questionnaires, we computed G-scores for our fMRS participants for subsequent analyses. After excluding fMRS data from participants (*n* = 4) who did not meet quality control criteria, Glx concentrations from the remaining participants were used in the current study. Participants performed decision-making tasks during MR scanning. Behavioral data collected through the two-armed bandit task were fitted to six candidate models, and the best-fitting model parameters (e.g., error sensitivity, decision temperature, and decay rate) were subsequently employed in the analysis. PHQ-9, patient health questionnaire 9; GAD-7, generalized anxiety disorder 7; STAI-X1, state-trait anxiety inventory X1; G-score, general psychopathological factor for anxiety and depression; Glx, glutamate complex; fMRS, functional magnetic resonance spectroscopy; RL, reinforcement learning; CRLB, Cramèr-Rao lower bounds.

By focusing on the AIC, this study aims to provide a more nuanced understanding of how glutamatergic activity contributes to the neurobiological basis of general psychopathology (Figure 1). These insights could have significant implications for the development of targeted interventions, particularly those that modulate glutamate signaling, in the treatment of anxiety and depression.

## 2 Methods

### 2.1 Participants

Fifty-six subjects (16 women, mean age 22±2 years) participated in this study. On average, participants scored 1.7±2.0 on the PHQ-9, 2.2±1.5 on the GAD-7, and 43.0±9.1 on the STAI-X1. All had normal or corrected-to-normal vision and normal stereo-acuity. Four subjects were excluded from analysis for one or more of the following reasons: early termination of the experiment, difficult MRS voxel placement as evidenced by T1-weighted image, and poor signal-to-noise ratio (SNR) in the metabolite spectra. Subsequently, the final data set comprised 52 subjects (12 women). Each participant took part in the MRS session. Volunteers received reimbursement of 50,000 KRW (≈40 USD). Participants first completed PHQ-9, GAD-7, and STAI-X1 questionnaires in a face-to-face session up to 2 weeks before their MRS scan (Hahn, 1996; Kroenke et al., 2001; Spitzer et al., 2006; Spielberger, 2010). This study was approved by the Institutional Review Board of KAIST. All participants provided informed written consent.

### 2.2 MR data acquisition

Magnetic resonance data were collected using a Siemens 3T Verio scanner (Erlangen, Germany) at KAIST. High-resolution T1-weighted structural images (MPRAGE, TR = 2,400 ms, TE = 2.02 ms, TI = 1,000 ms, flip angle: 8°, FOV: 224 × 224 mm, voxel size: 0.7 × 0.7 × 0.7 mm^3^) were acquired from the participants. After structural image acquisition, shimming was performed using FASTESTMAP (Gruetter and Tkáč, 2000), and water suppression pulses were calibrated for 1H-MRS. Single-voxel 1H-MRS using semilocalization by adiabatic selective refocusing (semi-LASER, TR = 3,000 ms, TE = 28 ms, 400 scans, 2,048 complex points) was acquired from mPFC VOIs (15 × 20 × 20 mm^3^) and AI VOIs (30 × 20 × 20 mm^3^), which a psychiatrist or a neurologist manually positioned. Because single-voxel fMRS cannot acquire multiple regions simultaneously, AIC and mPFC scans were run in separate sessions spaced 10–20 min apart, with the order of VOI acquisition randomized across participants to avoid order effects. The semi-LASEER sequence maintains a uniform B1 field and desired flip angle within each voxel (Zhu and Barker, 2010). Unsuppressed water signals were also recorded to enable eddy-current and phase correction during preprocessing.

### 2.3 Quality control of MRS data

The preprocessing pipeline for 1H-MRS data consisted of eddy current correction, frequency correction using a cross-correlation algorithm, and phase correction using the least square algorithm, part of MRspa software (version 1.5 f) (Deelchand et al., 2018). To determine the CSF fraction within the VOI, the metabolite concentrations were estimated using LCModel software version 6.3-1J (Provencher, 1993) and a basis set (Deelchand et al., 2015; Park et al., 2018). The estimates with CRLB <20% were considered reliable (Ip et al., 2017). Seven of the metabolites met the CRLB criterion: NAA, NAAG, mI, glutamate, Glx, tCho, and tCr. Based on the visual inspection and preprocessing steps described above, we conducted a further investigation for quality control. We investigated the LCModel fitting SNR and motion parameters for the MRS data.

### 2.4 Anatomical consistency among participants' VOIs

Transformation matrices for each participant were acquired for the transformation of B0 to T1w images to the standard 1 mm^3^ MNI 152 space using Advanced Normalization Tools. Then, the transformation matrix transformed each VOI from the T1w space to the standard space. Finally, the generalized dice similarity coefficient as a measure of overlap was computed from the intersection and union of VOIs (Park et al., 2018; Kim et al., 2019). We used the Shen brain atlas (Shen et al., 2013), which consists of 268 regions of interest, including the AIC and mPFC. The generalized dice coefficient of the VOIs of 52 participants was 0.68 for the mPFC and 0.62 for the AIC (Supplementary Figure 10). Moreover, because a dice similarity coefficient of 0.6 or greater is regarded as an excellent agreement for similarities between pairs (Crum et al., 2006; Zou et al., 2004), our data can be considered reliable for anatomical consistency.

### 2.5 Two-armed bandit task with gain and loss outcomes

**Figure 2:**
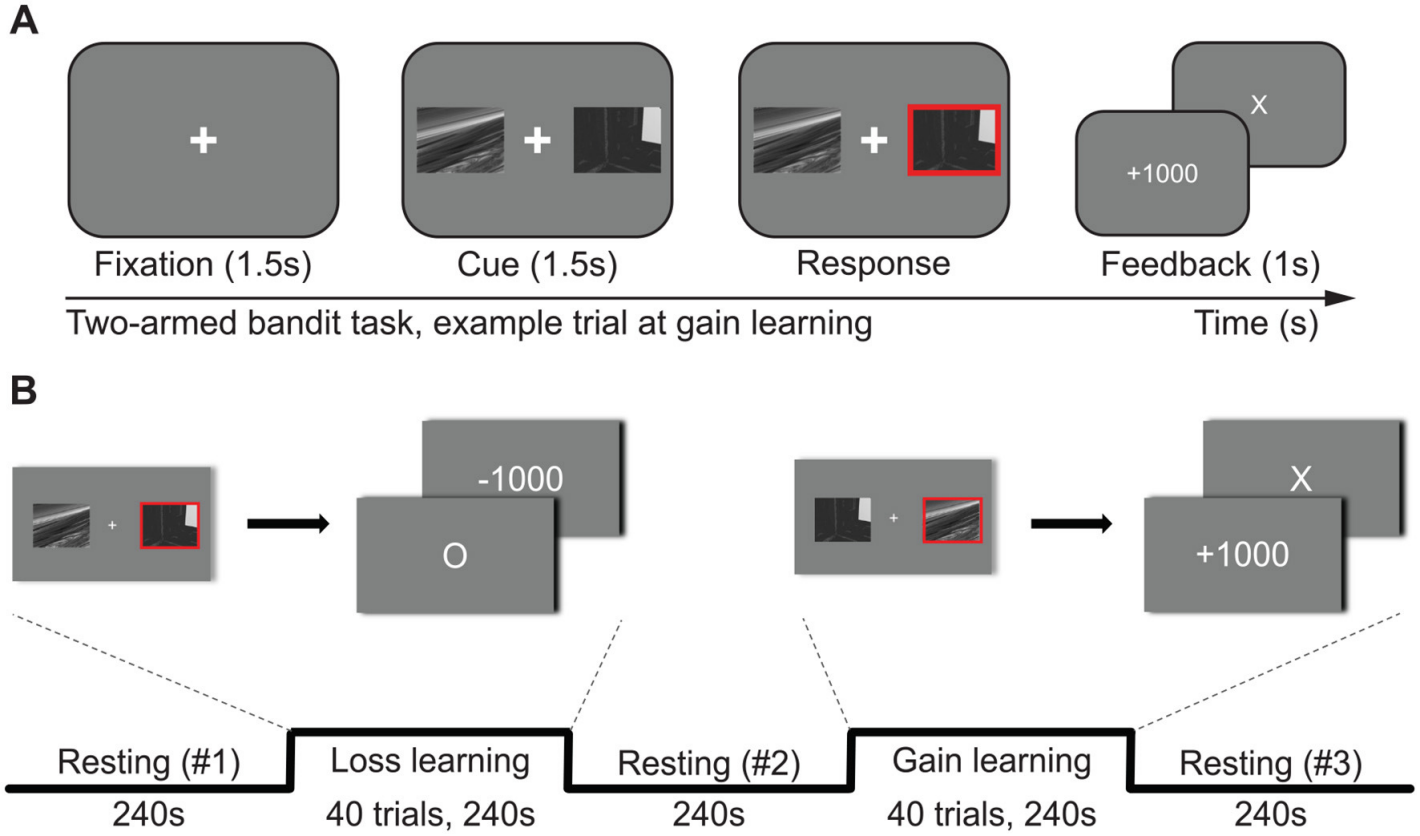
The experimental design. Participants completed two versions of a two-armed bandit task, one with gains and one with losses. In the gain block, the option with the higher reward probability yielded +1,000 points on 70% of trials and 0 points on 30% of trials, whereas the alternative option yielded the opposite probabilities. While learning from losses, the wrong option has a 70% chance of losing 1,000 points, and the right option has a 30% chance of no loss. **(A)** A trial consisted of fixation (~1.5 s)—cue (1.5 s)—outcome feedback (1 s), and the fixation interval ranged from 1.2 s to 1.8 s (mean 1.5 s) for jittering. Participants were instructed to respond as soon as possible; otherwise, the cue disappears and loses the trial. **(B)** Participants performed the loss-learning task and the gain-learning task sequentially. Each learning task consisted of 40 trials. Resting-state sessions were presented before, between, and after learning sessions. The resting session right before each task (resting #1 for loss learning and resting #2 for gain learning) was used as the baseline for assessing task-related neurochemical changes.

Subjects performed decision-making tasks during MR scanning. The subjects inferred the correct answer among two options in the decision-making task. The correct option gives a good outcome with a high probability (70%) and a bad outcome with a low probability (30%). The wrong answer gives a bad outcome with a high probability (70%) and a good outcome with a low probability (30%). Subjects performed two types of decision-making tasks (Figure 2). The first was the penalty task, which lost a score (–1,000) for a bad outcome and nothing (±0) for a good outcome. The second was the gain task, which gained a score (+1,000) for a good outcome and nothing (±0) for a bad outcome. Eighty trials were made on each of the two types of tasks. A 5,000 KRW (Korean currency, ≈4 USD) bonus was paid to participants who scored 8,000 or more. The outcome probability of each option was hidden from the subjects. The penalty task block preceded the gain task in all subjects.

On the day of scanning (all between 9 AM and 12 PM), they ran practice trials in the scanner control room—continuing until they reported stable performance on both gain and loss tasks—immediately before entering the scanner. Each participant completed at least one 40-trial practice block (20 gain, 20 loss). Practice blocks were repeated until the participant attained ≥50% correct (chance level) on both tasks. Participants who reached criterion could request additional practice blocks if they wished. This procedure ensured that behavior was stable before acquisition began. In the scanner, each participant completed 10 blocks in total: three resting-state blocks and, for each VOI (AIC and mPFC), one loss block and one gain block. Each task block contained 40 decision trials, yielding 160 trials overall (40 trials × 2 outcome types × 2 VOIs).

### 2.6 Reinforcement learning models

A total of six Rescorla-Wagner model variations were constructed. Model 1 assumes a one-armed bandit with an error sensitivity (ϵ).


$$\begin{array}{l} PE_{t}=outcome_{t}-v_{t,chosen};outcome\in \left\{0,1\right\},v\in \left[0,1\right]\\ \;v_{t+1,chosen}=v_{t,chosen}+\epsilon *PE_{t};\epsilon \in \left[0,1\right] \end{array}$$

The information update for an unchosen option was done simultaneously in the opposite direction.


$$v_{t+1,unchosen}=1-v_{t,chosen}$$

At the observation level, a decision was made by transforming values to the softmax function.


$$probability_{t,chosen}=\frac{e^{v_{t,chosen}}}{e^{v_{t,chosen}}+e^{v_{t,unchosen}}}$$

Model 2 assumes a two-armed bandit, which only updates the chosen option's value.


$$v_{t+1,unchosen}=v_{t,unchosen}$$

The decision temperature (τ) was added to the observation level in Model 3. Decision temperature controls the exploration-exploitation trade-off of human behavior by adjusting choice probability. As the decision temperature becomes small, choice selection becomes more random and shows exploration behavior, and choice selection becomes more deterministic and shows exploitation behavior in the opposite case.


$$probability_{t,chosen}=\frac{e^{\tau *v_{t,chosen}}}{e^{\tau *v_{t,chosen}}+e^{\tau *v_{t,unchosen}}};\tau \in \left[0,1\right]$$

The decay rate (λ) was added in Model 4, which resulted in the decay of the value of the unselected option.


$$v_{t+1,unchosen}=\text{λ}*v_{t,unchosen};\text{λ}\in \left[0,1\right]$$

Models 5 and 6 are modified versions of Models 3 and 4, respectively, in which the lapse parameter (*L*) replaces the decision temperature parameter (τ). The lapse parameter explains the exploration-exploitation trade-off (Aylward et al., 2019).


$$probability_{t,chosen}=\frac{e^{v_{t,chosen}}}{e^{v_{t,chosen}}+e^{v_{t,unchosen}}}*\left(1-L\right)+L/2;L\in \left[0,1\right]$$

The optimal learning model was selected using the leave-one-out information criterion (*LOOIC*), which gauges the pointwise prediction accuracy for new data based on a Bayesian model This approach calculates the log-likelihood of each observation under posterior simulations of the model parameters, providing a robust estimate of the model's predictive performance (Vehtari et al., 2017). The model with the lowest *LOOIC* is considered the best, as it indicates the model has the best trade-off between fitting the data well and being parsimonious.

### 2.7 Baron and Kenny's four-step approach to mediation

To assess the behavioral parameters' role in mediating between neurochemicals and the mental state, we first performed Baron and Kenny's four-step mediation analysis (Baron and Kenny, 1986). Step one was to regress the dependent variable on the independent variable to confirm that the independent variable is a significant predictor of the dependent variable. Step two was to regress the mediator on the independent variable to ensure that the independent variable was a significant predictor of the mediator. There will be no mediation if the mediator is not associated with the independent variable. Finally, step three was to regress the dependent variable on both the mediator and independent variable to confirm that the mediator is a significant predictor of the dependent variable, and the strength of the coefficient of the previously significant independent variable in step one is now significantly reduced.

### 2.8 Statistical analysis

All statistical tests were conducted in R (version 4.4). Behavioral performance (accuracy, win-stay/lose-stay) was examined with one-sample and paired-sample *t*-tests against chance levels. Time effects on Glx and on resting-state comparisons were evaluated using one-way repeated-measure ANOVA with Greenhouse-Geisser correction for nonsphericity, followed by Bonferroni-corrected *post hoc* pairwise contrasts. Correlations between metabolites, model parameters, and questionnaire scores employed Pearson's or Spearman's rank correlations, with Bonferroni adjustment for multiple comparisons. Mediation analyses followed Baron and Kenny's four-step regression approach, and moderation was tested via linear regression including an interaction term between Glx and error sensitivity. All tests used a two-tailed α = 0.05.

## 3 Results

### 3.1 Behavioral results

Two-armed bandit tasks with gain and loss outcomes were performed (Figure 2). The correct rates of the two-armed bandit tasks of 52 subjects were above the chance rate for both learning from gains (62.0%, *p* < 0.001) and learning from losses (60.9%, *p* < 0.001). Performance did not differ by the learning type (*p* = 0.142); however, the win-stay rate (WS), which represents a tendency to remain in the current choice when they win the game, was higher than the lose-stay rate (LS) for both gain outcome (WS: 84.5%, LS: 79.3%, *p* = 0.003) and loss outcome (WS: 85.4%, LS: 80.7%, *p* = 0.003).

### 3.2 Computational modeling results

A total of six models were fitted to the data, and the optimal learning model was selected using the leave-one-out information criterion (*LOOIC*) (see reinforcement learning models in the Materials and methods section). The winning model was the *Rescorla-Wagner* model with three parameters: error sensitivity, decision temperature, and decay rate (Model 4 in Table 1). Parameters were successfully recovered (Supplementary Figure 2), meaning that the model sufficiently fitted parameters despite the innate correlation between error sensitivity and decision temperature (Addicott et al., 2021).

**Table 1:** Model specification and fit indices.

**RL models**	**Error sensitivity**	**Decision temperature**	**Lapse**	**Decay rate**	***LOOIC***
**One-arm**					
Model 1	✓	–	–	–	9,156
**Two-arm**					
Model 2	✓	–	–	–	7,377
Model 3	✓	✓	–	–	7,317
Model 4	✓	✓	–	✓	6,330
Model 5	✓	–	✓	–	7,448
Model 6	✓	–	✓	✓	7,510

Model 4 (error sensitivity + decision temperature + decay rate) showed the lowest LOOIC. The details of each model are described in the Methods' reinforcement learning model section. RL, reinforcement learning; LOOIC, leave-one-out information criterion, lower values indicate better fit.

### 3.3 Spectra fitting for glutamate-glutamine complex

**Figure 3:**
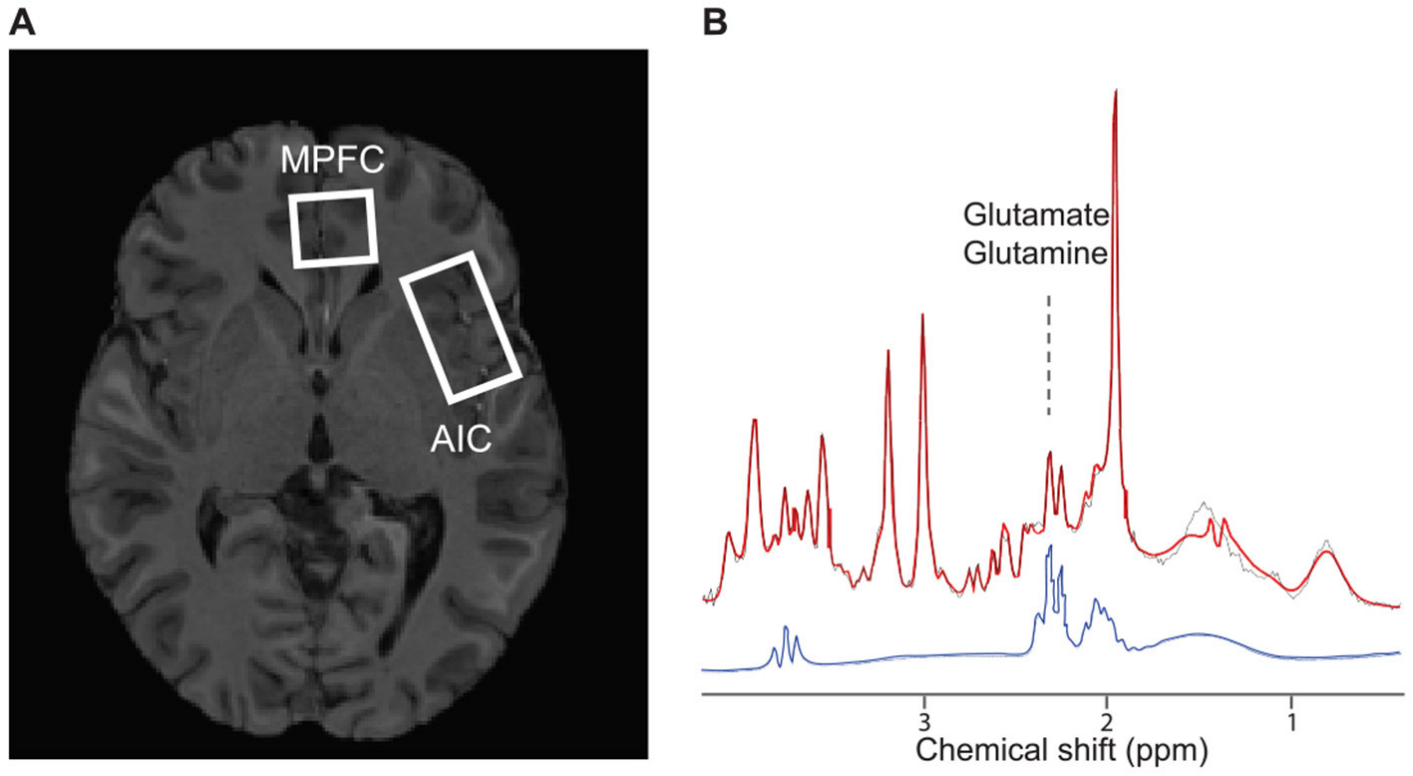
The volume of interest and spectroscopic data sample. **(A)** Axial cross-section of the single-subject representative voxel locations for the left anterior insular and medial prefrontal cortex were superimposed on the anatomical scan from the same subject. **(B)** The cortical metabolic profile by 1H-fMRS analysis. Profile shows the average of 80 spectra acquired during the first resting period. The red and black line depicts the raw and fitted signals, respectively. The blue line is an isolated signal for Glx. AIC, anterior insular cortex; mPFC, medial prefrontal cortex; MRS, magnetic resonance spectroscopy.

The LCModel fit characteristics for metabolites from each volume-of-interest (VOI, Figure 3A), including Glx, and a representative spectrum, are depicted in Figure 3B. After spectra preprocessing, which included averaging, frequency, and phase correction using MRspa software (version 1.5 f) (Deelchand et al., 2018), the metabolite concentrations were estimated using LCModel software version 6.3-1J (Provencher, 1993). The analysis incorporated a basis set (Deelchand et al., 2015; Park et al., 2018) and accounted for the cerebrospinal fluid (CSF) fraction within the VOI. Metabolite estimates with Cramèr-Rao lower bounds (CRLB) <20% were considered reliable. Seven metabolites—N-acetylaspartate (NAA), N-acetylaspartylglutamate (NAAG), myo-inositol (mI), glutamate (Glu), glutamate+glutamine (Glx), glycerylphosphorylcholine+phosphorylcholine (tCho), and creatine + phosphocreatine (Cr + PCr) met the CRLB criterion.

### 3.4 Dynamic Glx changes during reinforcement learning

Glx concentration in each block was calculated with fMRS data acquired during reinforcement learning experiments, which consisted of resting-loss-resting-gain-resting blocks. A full model of the AIC (Montreal Neurological Institute (MNI) coordinates: *x* = −38.2, *y* = 8.9, *z* = −3.9) Glx using one-way repeated measures ANOVA correcting for nonspherical distribution showed a significant time effect [*F*_(4,47)_ = 2.63, *p* = 0.036]. *Post hoc* analysis with Bonferroni correction did not reveal significant differences between blocks. Conversely, Glx levels in the mPFC (*x* = 0.8, *y* = 47.0, *z* = −2.0) did not show a significant time effect [*F*_(4,47)_ = 1.62, *p* = 0.169]. These results were consistent even when Glx was referenced to the Cr+PCr level. Our findings suggest that Glx concentration in the AIC, but not the mPFC, decreased during or after reinforcement learning tasks.

### 3.5 Post-learning decrease in resting AIC Glx

To test whether the significant time effect on resting-state Glx specifically reflected an after-effect of the gain learning task, we compared AIc Glx at three resting intervals—pre-task baseline, between the loss and gain blocks, and post-task—using a one-way repeated measures ANOVA with Greenhouse-Geisser correction for nonsphericity. This analysis revealed a significant main effect of time in the AIC [*F*_(2,49)_ = 3.54, *p* = 0.033] but not on the mPFC [*F*_(2,49)_ = 1.63, *p* = 0.200]. Bonferroni-corrected *post hoc* tests showed that resting AIC Glx decreased from the between-task to the post-task interval (Δ = −2.27%, *p* = 0.045), indicating a specific post-gain-learning reduction in Glx. These findings held when normalizing to Cr + PCr (Supplementary Figure 3), with a significant time effect in the AIC [*F*_(2,49)_ = 4.15, *p* = 0.019], and a consistent post-task drop (Δ = −2.56%, *p* = 0.022), but no effects in the mPFC [*F*_(2,49)_ = 0.438, *p* = 0.646].

### 3.6 Task-specific AIC Glx decrease during gain learning

**Figure 4:**
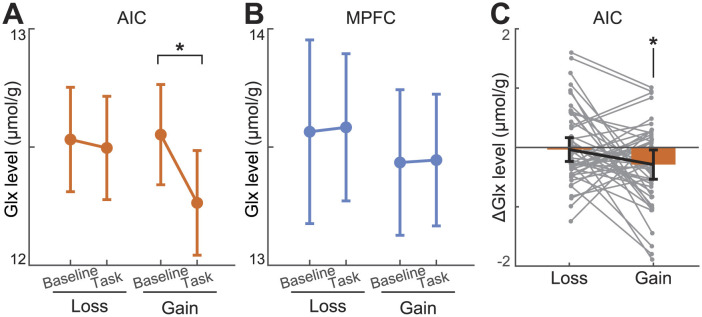
Glutamate changes during learning tasks. **(A, B)** During gain learning, Glx levels decreased in the AIC but not in the mPFC. Glx did not show significant changes during loss learning. The baseline refers to the resting period immediately before performing each task. **(C)** AIC Δ Glx shows that it has dropped during gain learning, but not loss learning. ΔGlx refers to task block Glx minus preceding resting-state Glx. The * indicates that the time effect of the partial ANOVA model was significant at the level of *p* < 0.05. Error bars indicate 95% confidence intervals.

We tested whether the Glx level changes during learning compared to the baseline Glx level. Baseline(pre-task), during-task, and post-task Glx levels were entered into a one-way repeated measures ANOVA. Learning from the gain model with AIC Glx had a significant task effect [*F*_(2,49)_ = 4.27, *p* = 0.017; Figures 4A, C]. The *post hoc* comparisons after the Bonferroni correction revealed that the Glx level during learning from gains was significantly decreased compared to that at baseline (Δ = −2.21%, *p* = 0.034). This decrement was not restored after the task (Δ = +0.18%, *p* = 0.927). AIC Glx during learning from losses had no significant task effect [*F*_(2,49)_ = 0.289, *p* = 0.750]. Also, Glx concentration in the mPFC had no significant task effect both on learning from gains [*F*_(2,49)_ = 1.17, *p* = 0.313] and losses [*F*_(2,49)_ = 0.402, *p* = 0.670; Figure 4B]. These results held when normalizing Glx to Cr+PCr (Supplementary Figure 3). Learning from gains repeated measures ANOVA, which used AIC Glx corrected to Cr+PCr, was significant [*F*_(2,49)_ = 8.75, *p* < 0.001], and the Glx level decreased during learning (Δ = −3.66%, *p* < 0.001), and was not restored after learning (Δ = +1.15%, *p* = 0.222). Further, we used absolute concentrations rather than the normalized to Cr+PCr.

### 3.7 Associations between Glx and computational parameters

**Figure 5:**
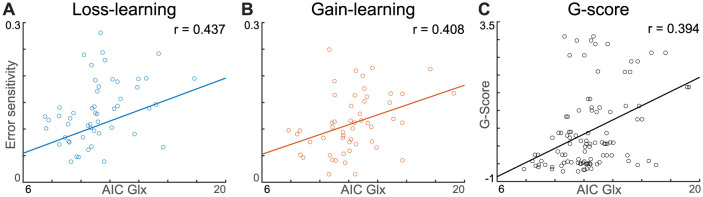
Pearson correlation between AIC Glx, error sensitivity, and G-score. **(A, B)** Both loss- and gain-error sensitivity show significant Pearson correlation with AIC Glx. **(C)** G-score shows a significant linear correlation with AIC Glx. The depicted lines are least-squares lines.

Pearson's correlation analyses were performed to investigate the association between each behavioral parameter acquired from the winning model with Glx level and learning-induced Glx shift (ΔGlx, compared to baseline). Highlighting the role of Glx on behavior, baseline AIC Glx, but not ΔGlx, showed a significant relationship with error sensitivity to losses (*r* = 0.437, *p* = 0.002) and gains (*r* = 0.408, *p* = 0.005). Furthermore, each error sensitivity was significantly correlated with the Glx concentration in the corresponding task block (Figures 5A, B). However, there was no relationship between baseline AIC Glx and decision temperatures (|*r*| < 0.085, *p* > 0.594) or decay rates (|*r*| < 0.241, *p* > 0.115). Furthermore, AIC ΔGlx did not have a relationship with the corresponding task error sensitivity (loss: *r* = 0.180, *p* = 0.201, gain: *r* = 0.076, *p* = 0.592), decision temperature (loss: *r* = −0.032, *p* = 0.824, gain: 0.071, *p* = 0.620), and decay rate (loss: *r* = 0.068, *p* = 0.633, gain: *r* = −0.113, *p* = 0.426). In the mPFC, behavioral parameters of both gain and loss conditions did not significantly correlate with either baseline Glx (|*r*| < 0.229, *p* > 0.103) or ΔGlx (|*r*| < 0.215, *p* > 0.126). Pearson's correlation was used, and significance levels were corrected with Bonferroni methods. The positive association between AIC Glx and error sensitivity existed even though AIC Glx had significantly decreased during learning from the gain.

### 3.8 Calculation of the general psychopathology score reflecting depression and anxiety severity: the bifactor analysis

Since previous studies have reported high comorbidity between anxiety and depression (Gorman, 1996), the association with glutamatergic metabolites of anxiety and depression (Nasir et al., 2020) may not be independent. In this study, we calculated the shared score of anxiety and depression severity from Patient Health Questionnaire 9 (PHQ-9) (Kroenke et al., 2001), General Anxiety Disorder 7 (GAD-7) (Spitzer et al., 2006), and State-Trait Anxiety Inventory X1 (STAI-X1) (Spielberger, 2010; Hahn, 1996) using the bifactor analysis. With exploratory (*n* = 17,413) and confirmatory (*n* = 13,450) bifactor analyses in a large independent sample of the general population, a bifactor model was established for calculating the shared score of anxiety and depression, termed as a general factor score (G-score) here (Supplementary Table 1). In the result of the model fit, the bifactor model showed the best fit in chi-square tests (Supplementary Table 2). Items from the six subgroups loaded similarly and strongly onto their group factors (Supplementary Figure 1). In addition, we computed “omega hierarchical” reliability, which denotes the proportion of variance in a total sum score attributable to the general factor. Our bifactor model's hierarchical and total omega scores were 0.72 and 0.97, respectively. The bifactor analysis is described in detail in the Supplementary Method. Chi-square test results showed the superiority of the orthogonal bifactor model (Supplementary Table 2), supporting the computation of a total score and interpreting it as a reflection of the general factor of anxiety and depression (Bornovalova et al., 2020) primarily.

### 3.9 G-score is associated with AIC Glx

PHQ-9 (*r* = 0.409, *p* = 0.003), STAI-X1 (*r* = 0.357, *p* = 0.010), and GAD-7 (*r* = 0.300, *p* = 0.033) had a positive relationship with baseline (pre-task resting) AIC Glx. Spearman's rank correlation, a nonparametric correlation measure, was used because the survey scores did not satisfy the assumption of normality. The general factor score of anxiety and depression also showed a significant correlation with AIC Glx at baseline (*r* = 0.394, *p* = 0.004, Figure 5C) and with the average AIC Glx during the whole task (*r* = 0.411, *p* = 0.002, Supplementary Table 3). However, AIC ΔGlx was unrelated to the g-score (loss: *r* = 0.107, *p* = 0.452, gain: *r* = −0.018, *p* = 0.897). Multiple linear regression was performed using a G-score with independent variables of baseline AIC Glx, mPFC Glx, age, and sex. A significant regression predictor was found [*F*_(4,47)_ = 3.92, *p* = 0.007], with an adjusted *R*^2^ of 0.25. AIC Glx was a significant predictor of the G-score (*b* = 0.183, *p* = 0.004), whereas mPFC Glx was not (*b* = 0.004, *p* = 0.969). All reported *p*-values were Bonferroni corrected.

### 3.10 Mediation analysis: higher error sensitivity mediated positive relation between Glx and G-score

**Figure 6:**
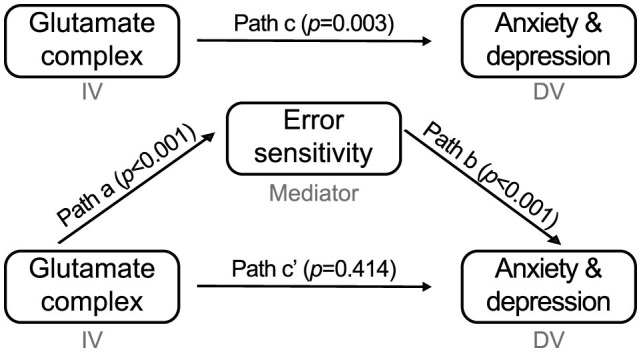
Parameter estimate for the four-step mediation analysis. AIC Glx (independent variable) predicts G-score for anxiety and depression (dependent variable). After controlling for the error sensitivity (mediator), those effects were no longer significant.

To assess the error sensitivity's mediation effect on the association between baseline Glx concentration in the AIC and G-score, we performed Baron and Kenny's four-step mediation analysis (Figure 6). Regression analysis predicting the G-score (dependent variable, DV) using AIC Glx (independent variable, IV) showed a significant association (*b* = 0.409, *p* = 0.003), which corresponds to step one in the mediation analysis (IV → DV). In step two, we regressed the average error sensitivity (mediator) on the Glx level, which was a significant predictor of error sensitivity (IV → mediator; *b* = 0.437, *p* < 0.001). Finally, we regressed the G-score using error sensitivity and the Glx level in step three (mediator → DV). The error sensitivity was a significant predictor of the G-score (*b* = 0.762, *p* < 0.001). At the same time, the effect of Glx was no longer significant (IV → DV, controlling for mediator; *b* = 0.081, *p* = 0.426), implying that error sensitivity mediates a positive association between AIC Glx and the general psychopathological factor score of anxiety and depression.

### 3.11 Moderation analysis: error sensitivity did not modulate the relationship between Glx and G-score

Whereas mediation tests whether error sensitivity explains how Glx influences the G-score, moderation examines whether the strength of the Glx-G-score association depends on levels of error sensitivity. We ran a linear regression with the G-score as the outcome, AIC Glx as the predictor, and error sensitivity as the moderator. The Glx × error sensitivity interaction was not significant (*b* = 0.828, *p* = 0.223). Although the full model was significant (*F* = 29.2, *p* < 0.001), neither AIC Glx (*b* = −0.175, *p* = 0.446), error sensitivity (*b* = 0.084, *p* = 0.881), nor their interaction predicted the G-score. Removing the interaction term yielded a significant model (*F* = 42.62, *p* < 0.001) in which error sensitivity predicted the G-score (*b* = 0.762, *p* < 0.001), but AIC Glx did not (*b* = 0.081, *p* = 0.406).

### 3.12 Specificity of Glx associations relative to other metabolites

To test whether the relationship between AIC Glx and error sensitivity reflected a general metabolic effect rather than Glx-specific signaling, we repeated the same Pearson correlation analyses for six other metabolites with CRLB < 20% (NAA, NAAG, mI, Glu, tCho, Cr + PCr). None of these metabolites correlated significantly with error sensitivity to gains or losses (all |*r*| < 0.18, *p* > 0.20) or with learning-induced spectral changes (all |*r*| < 0.15, *p* > 0.25). These null results support the specificity of the Glx-error sensitivity associations.

## 4 Discussion

The present findings provide convergent evidence that the anterior insula cortex (AIC) glutamate-glutamine complex (Glx) levels are associate with individual differences in error sensitivity during reinforcement learning and correlated with a trandiagnostic dimension of depression and anxiety. Specifically, baseline AIC Glx was positively related to both gain- and loss-related error sensitivity, suggesting that higher excitatory tone in this region is linked to an amplified perception of prediction errors. Such overestimation of errors is often observed in internalizing disorders, where individuals tend to focus disproportionately on negative outcomes and worrisome predictions. Moreover, this behavioral overestimation of errors statistically mediated the association between AIC Glx and a unified factor of anxiety and depression. Clinically, these results imply that insular glutamatergic processes contributing to error processing may underlie a core vulnerability across anxiety and depressive symptoms, and thus could inform future efforts to refine diagnostics or tailor interventions.

A notable finding is that Glx changes during reinforcement learning were specific to the AIC and not evident in the medial prefrontal cortex (mPFC), despite the well-established role of the mPFC in mood regulation. One possible interpretation is that AIC and mPFC subserve partially distinct functions within broader corticolimbic circuits implicated in affective processing. The AIC is often described as a key region for interoception, salience processing, and the integration of bodily signals into subjective feelings (Craig, 2009; Ullsperger et al., 2010). By this account, fluctuation of insular Glx levels may reflect dynamic shifts in the evaluation of salient prediction errors, particularly during reward-based tasks such as learning from gains. In contrast, the mPFC, especially its ventromedial portion, is frequently associated with long-term mood regulation, self-referential thought, and decision-making guided by more abstract values (Alexander and Brown, 2011). The lack of observable task-related changes in mPFC Glx could mean that the mPFC's involvement in mood regulation is driven by more enduring neurochemical or functional changes rather than the short-term fluctuations revealed by our reinforcement learning paradigm (Billeke et al., 2020). Alternatively, the mPFC may exhibit more subtle neurometabolic shifts or might rely on other neurotransmitter systems during acute learning, making the immediate Glx response more challenging to detect without specialized methods or larger sample sizes. The dissociation underscores the possibility that the AIC is preferentially responsible for the on-the-fly registration of error-related signals, whereas the mPFC might orchestrate broader cognitive-emotional integrations over longer intervals.

Another relevant observation is that although AIC Glx decreased specifically during gain-based tasks, the trait-like association between baseline Glx and error sensitivity remained robust. This pattern implies that while learning-related processes may transiently lower local glutamate levels—perhaps reflecting increased metabolic demand or altered excitatory signaling during the task—such an acute shift does not disrupt the more stable, baseline neurochemical context that correlates with trait-level biases in error processing. These findings also fit into the broader framework that, although insular metabolic activity fluctuates with momentary salience detection and prediction error, baseline Glx indices may represent a more enduring predisposition toward negative affect and cognitive distortion. The novelty and importance of this study lie in its demonstration that a neurochemical marker in the AIC can be mechanistically linked to a transdiagnostic dimension of depression and anxiety via error sensitivity. While prior work has associated insular activity and glutamatergic metabolites with either anxiety or depression in isolation (Nasir et al., 2020), the emphasis here on a bifactor model clarifies that a shared internalizing dimension underpins these conditions. Moreover, the mediation by reinforcement learning's error sensitivity parameter suggests a specific cognitive pathway—overestimation of errors—through which elevated AIC Glx confers risk for internalizing psychopathology. These insights may promote more targeted approaches in computational psychiatry, emphasizing interventions that modulate insular function or attenuate error overvaluation. For example, real-time neurofeedback methods could focus on downregulating hyperactivation in the insula during error detection, or pharmacological tools could aim at modulating glutamatergic signaling in this region to reduce maladaptive salience attribution.

Despite its strengths, a primary limitation of the current study is its relatively modest sample size for functional MRS investigations, which can raise questions about statistical power, particularly for detecting more nuanced individual differences. Furthermore, the cross-sectional design does not definitively establish that elevated AIC Glx causes increased error sensitivity and subsequent anxiety-depression; it remains plausible that chronic negative affect gradually alters insular glutamate metabolism or that a bidirectional relationship exists. Another point to consider is that we focused on two targeted regions—the AIC and mPFC—and thus cannot rule out the possibility that other neural circuits or neurotransmitter pathways contribute to the observed effects. Additionally, MRS signals can be sensitive to partial volume effects, scanner hardware, and data processing methods, so efforts to replicate these findings at higher field strengths or with more refined voxel placement are necessary. Finally, because the loss block always preceded the gain block—even though participants completed extensive practice trials to familiarize themselves with both tasks—we cannot fully exclude order or adaptation effects that might have influenced Glx dynamics; future studies should counterbalance block order to disentangle task-specific neurochemical changes from sequence-related confounds (Garrett and Daw, 2020; Dundon et al., 2020). Moreover, Because scheduling constraints required some participants to finish the questionnaires 7–14 days before the MRS session, state-related fluctuations may have introduced additional noise. Although the PHQ9, GAD7, and STAI-X1 show robust test–retest reliability across similar intervals (Kroenke et al., 2001; Spitzer et al., 2006; Spielberger, 2010), the temporal gap remains a limitation. Future studies should collect psychometric and neuroimaging data on the same day—or use repeated questionnaire administrations—to quantify intra-individual variability Lastly, the selected bifactor model indicated that two group factors had higher values than their corresponding specific factors. Therefore, it is important to note that the specific factors with lower values (e.g., generalized anxiety) may still contribute to the general factor score calculated from the model, albeit to a lesser extent. This limitation suggests that caution should be exercised when interpreting the calculated scores, as the lower values of these specific factors may indicate a lack of meaningful variance. While the use of a bifactor model is an elegant way to distill the core dimension of anxiety-depression, future studies would benefit from assessing more clinically severe samples or adding other relevant constructs like worry or anhedonia to further validate and refine the transdiagnostic scope.

## 5 Conclusion

These results enrich our understanding of how the anterior insula's glutamatergic environment shapes not only the acute processes involved in detecting and weighting errors but also the broader affective landscape of depression-anxiety. The specificity of the AIC Glx changes, in contrast to a relative absence of such changes in the mPFC, suggests that the anterior insula may be particularly instrumental in driving momentary salience- and error-related computations, which, when exaggerated, predispose individuals to internalizing psychopathology. By bridging computational modeling, neurometabolic imaging, and advanced psychometric analyses, this study supports a model in which heightened AIC glutamate promotes the overestimation of prediction errors, thereby reinforcing anxious or depressive thought processes. Further longitudinal and interventional work will help ascertain whether modulating insular glutamate could attenuate these maladaptive error sensitivities and reduce the burden of internalizing disorders.

## Data Availability

The raw data supporting the conclusions of this article will be made available by the authors, without undue reservation.
