# Supplementary material for: Anterior insular cortex glutamate-glutamine (Glx) levels predict general psychopathology via heightened error sensitivity
Source: Front Neurosci. 2025 Jul 16;19:1592015. doi: 10.3389/fnins.2025.1592015 (PMC12307414; doi:10.3389/fnins.2025.1592015)
Supplement: Supplementary file 1 [file Table_1.docx]

**Supplementary Materials**

*Dataset used for bifactor analysis to calculate general psychopathological factor score*

Young adults aged 18 and 34 were included from the four centers in the Research Consortium for Young Adulthood Depression. The participants underwent medical examinations, including mental health measurements, and were enrolled in the study between January 1, 2018, and December 31, 2019. The research protocol for the present study was approved by the Korea Advanced Institute of Science and Technology (KAIST) Institutional Review Board.

Self-report questionnaires and other clinical data from four independent institutions were obtained from Center 1 (KAIST, n=17,322), Center 2 (Gachon University Hospital, n=69), Center 3 (Samsung Medical Center, n=91), and Center 4 (Seoul National University, n=14,238).

| **Dataset** | **Total** |
| --- | --- |
| **Center/**  **item** | **Centers 1+2+3+4**  (*n*=31,720) |
| Age (years)^*^ | 23.64 $\pm$3.96 (18-34) |
| Gender (Male)^**^ | 21,639 (68.22%) |
| MaDE | 102 (0.32%) |
| PHQ-9^*^ | 2.23 $\pm$3.42 (0-27) |
| GAD-7^*^ | 1.46 $\pm$2.77 (0-21) |
| STAI-S^*^ | 38.23 $\pm$8.69 (20-80) |

Supplementary Table 1. Basic demographic details of participants who provided questionnaire data. Please see our previous article for more details (Choi et al., 2021).

*Exploratory bifactor analysis*

An exploratory bifactor analysis was conducted on item-level responses to the PHQ-9, GAD-7, and STAI-X-1 in 17,413 of both Center 1 and Center 3. To determine the number of dimensions to use in the bifactor analysis, a parallel analysis was conducted using the ‘fa.parallel’ function from the Psych package in R. The parallel analysis compared the sequence of eigenvalues from the data to their corresponding eigenvalues from a random normal matrix of equivalent size (Floyd & Widaman, 1995; Humphreys, 1975). This procedure showed a seven-factor solution for our exploratory dataset.

We first conducted an oblique factor analysis; following this, we conducted a higher-order factor analysis on the lower-order factor (i.e., a general factor). This procedure is known as Schmid-Leiman (SL) orthogonalization (Schmid & Leiman, 1957). Using the ‘omega’ function from the Psych package in R, we rotated the factors obliquely and then did a Schmid-Leiman (SL) orthogonalization (Schmid & Leiman, 1957). These procedures produced SL loadings of one general factor and six specific ones. Finally, factor scores for each participant in the exploratory dataset were calculated using the Anderson-Rubin method (Anderson & Rubin, 1956), which maintains the orthogonality of the general and specific factor scores.

*Confirmatory bifactor analysis*

To test the reproducibility of the factor structure estimated in the exploratory bifactor analysis, we used a total of 13,450 in the dataset from both center 2 and center 4 after excluding 767 participants having missing data. For the confirmatory bifactor analysis, all items of three questionnaires were allowed to load on the general factor (Supplementary figure 1). Each item was also allowed to load onto a specific factor among the six ones. The assignment to the specific factor was determined when the item had a loading greater than 0.2 on that specific factor in the exploratory bifactor analysis. Ten items of STAI-S had loadings greater than 0.2 on the first specific factor, and the rest 10 of STAI-S on the third one. All seven items of GAD-7 were in the second factor. Among PHQ-9 items, the fourth factor consisted of 4, the fifth was 2, and the sixth was 3 items. After item assignment, factor loadings were re-estimated using diagonally weighted least squares estimation. This analysis was conducted using ‘cfa’ and ‘inspect’ functions from the Lavaan package in R. When applying the analysis to the MRS sample, factor loadings for the MRS sample are similar to those for the larger population (spearman rho = 0.752, cosine similarity = 0.980).


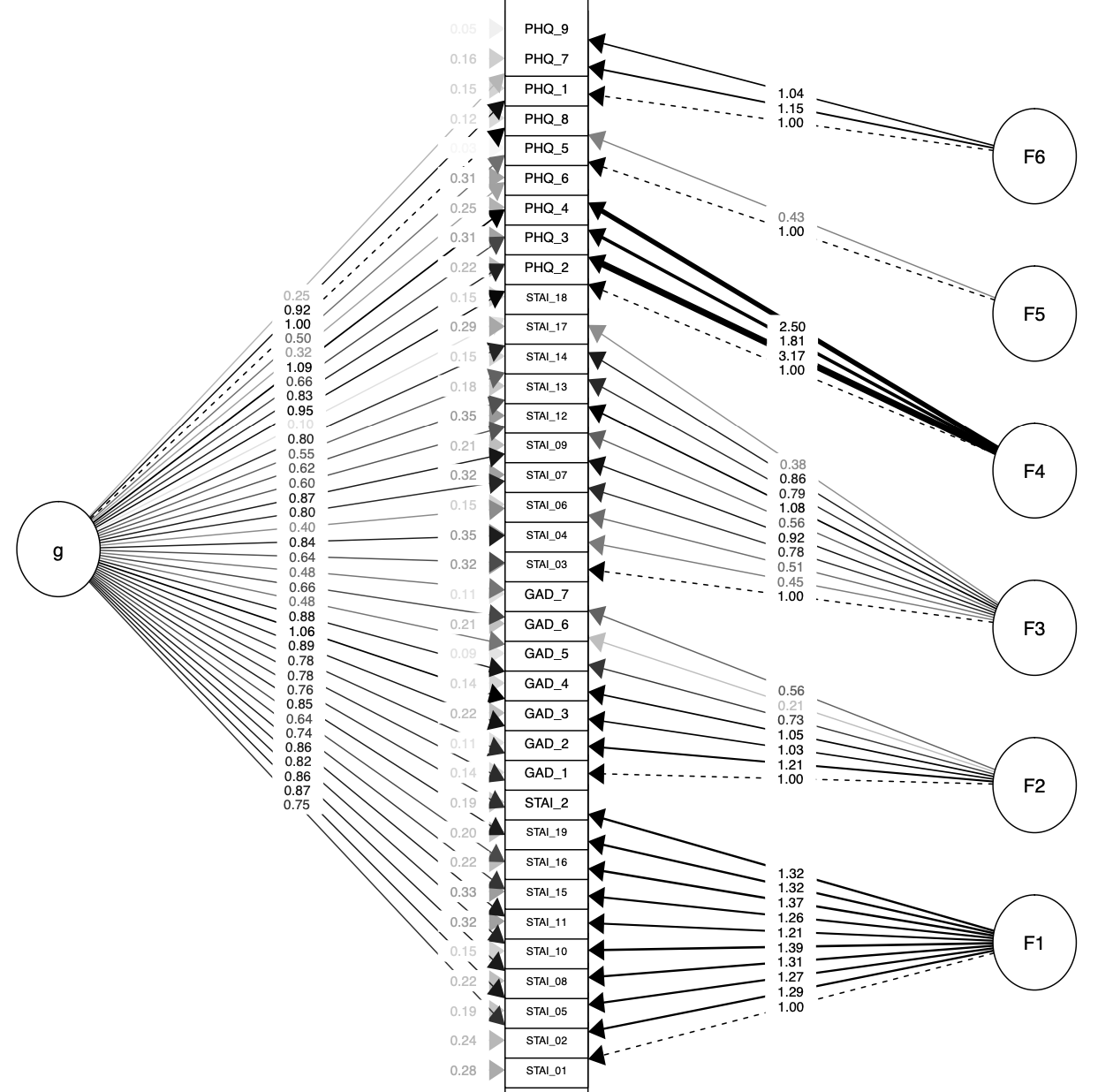


Supplementary Figure 1. Bifactor models fit to measures. Standardized factor loadings and residual variances are shown. Items from the six subgroups load similarly and strongly onto their group factors.

*Additional factor analysis for the comparison of the fit of the bifactor model*

To compare the fit of the confirmatory bifactor model, three other models were also applied to the total of 13,540 in the confirmatory dataset: a general factor-only model, a higher-order factor model, and a correlated factor model. For the general factor-only model, assignments of all items onto the general factor remained, while assignments for the specific factors were excluded. Conversely, the higher-order factor model excluded general factor assignments and used the same specific-factor assignments as the bifactor model. The correlated factor model includes correlated group factors but no overarching general factor. Quality of fit was determined by the comparative fit index (CFI), chi-square, Tucker-Lewis index (TLI), Akaike information criterion (AIC), and the root mean square error of approximation (RMSEA). These analyses were conducted using the Lavaan package in R. Finally, chi-square tests were performed to compare the fit of the bifactor model to three other factor models and showed the superiority of the orthogonal bifactor model (Supplementary Table 2).

In the bifactor analysis with six specific factors, we found that two group factors had higher values than their corresponding specific factors, suggesting they capture significant overlapping variance with the general factor. To simplify the model, we created a reduced version with four specific factors (all 9 PHQ items were merged into a factor). In this reduced model, one group factor also had a higher value than its specific factor. We compared both models using the ANOVA function from the psych package, revealing that the six-factor model had a higher general factor value along with lower AIC and BIC (Supplmentary Table 3), indicating a better fit to the data. Although the general factor score reflects shared variance among all specific factors, it can still be influenced by those with lower values. This means that while the general factor captures significant variance, the specific factors with lower values contribute to the overall construct, albeit to a lesser extent. Ultimately, we chose the six-factor model for further investigation of its association with Glx concentration in MRS.

In Supplementary Figure 1, the general factor was not dominated by just a few item and its loadings were even across indicators. Moreover, loadings of specific factors show very strong loadings (.50 or above) except 4 items (loadings of PHQ_8 in factor 5, STAI_18 and STAI_4 of F3, GAD_6 of F2 are 0.43, 0.38, 0.45, 0.21, respectively) onto the general factor, suggesting that it is a major factor that must be explained to understand general psychologic &/or psychiatric distress. Omega hierarchical which denotes the proportion of variance in a total sum score attributable to the general factor (Bornovalova et al., 2020). In the present study, omega hierarchical is 0.670 suggesting primarily reflecting the general factor score.

| Model | Free parameters | *X*^2^ | *X*^2^ diff | RMSEA | RMSEA 90% CI | CFI | TLI | AIC |
| --- | --- | --- | --- | --- | --- | --- | --- | --- |
| Orthogonal bifactor model | 108 | 31744.103 |  | 0.064 | 0.064-0.065 | 0.897 | 0.884 | 695595.49 |
| General factor-only model | 72 | 122983.445 | *X*^2^(36)=91239,  P < 0.001 | 0.123 | 0.123-0.124 | 0.598 | 0.573 | 786762.832 |
| Correlated factors model | 87 | 32028.153 | *X*^2^(21)=284, P < 0.001 | 0.063 | 0.063-0.064 | 0.897 | 0.888 | 695837.54 |
| Higher order factor model | 77 | 36022.354 | *X*^2^(31)=4278.3  P < 0.001 | 0.067 | 0.066-0.067 | 0.884 | 0.875 | 699811.742 |

Supplementary Table 2. Model fit indices for factor analyses

|  | total | general | group |  | df | AIC | BIC | Chisq | Chisq diff | RMSEA | df diff | Pr(>Chisq) |
| --- | --- | --- | --- | --- | --- | --- | --- | --- | --- | --- | --- | --- |
| Selected model | | |  |  | 537 | 685195 | 686164 | 21302 |  |  |  |  |
| g | 0.986 | 0.754 | 0.145 |  |  |  |  |  |  |  |  |  |
| F1 | 0.902 | 0.691 | 0.212 |  |  |  |  |  |  |  |  |  |
| ***F2*** | ***0.972*** | ***0.312*** | ***0.659*** |  |  |  |  |  |  |  |  |  |
| ***F3*** | ***0.901*** | ***0.363*** | ***0.538*** |  |  |  |  |  |  |  |  |  |
| F4 | 0.945 | 0.807 | 0.137 |  |  |  |  |  |  |  |  |  |
| F5 | 0.598 | 0.594 | 0.004 |  |  |  |  |  |  |  |  |  |
| F6 | 0.789 | 0.628 | 0.161 |  |  |  |  |  |  |  |  |  |
|  |  |  |  |  |  |  |  |  |  |  |  |  |
| Reduced model | | |  |  | 548 | 686952 | 687838 | 23080 | 1778.8 | 0.109 | 11 | < 2.2e-16 |
| g | 0.984 | 0.79 | 0.153 |  |  |  |  |  |  |  |  |  |
| F1 | 0.917 | 0.866 | 0.051 |  |  |  |  |  |  |  |  |  |
| ***F2*** | ***0.972*** | ***0.302*** | ***0.67*** |  |  |  |  |  |  |  |  |  |
| F3 | 0.935 | 0.465 | 0.47 |  |  |  |  |  |  |  |  |  |
| F4 | 0.904 | 0.889 | 0.015 |  |  |  |  |  |  |  |  |  |

**Supplementary Table 3.** Specific factors showing higher group factor value than its specific factor as ***Italic bold***. In the selected model, each specific factor represent as follows. F1: state anxiety; F2: generalized anxiety; F3: self-confidence; F4: depressed mood ; F5: difficulties in cognition ; F6: less volition

**Supplementary Figures 2~10**


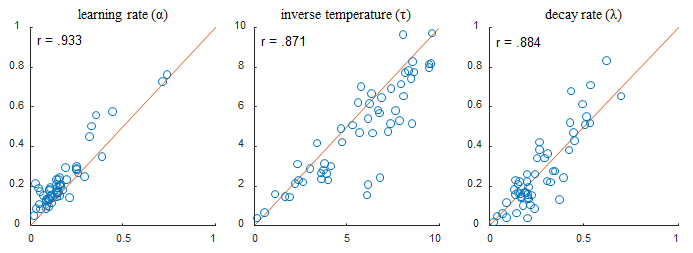


**Supplementary Figure 2.** Parameter recovery plot. Computed parameters had a significant positive correlation with simulated parameters.

*
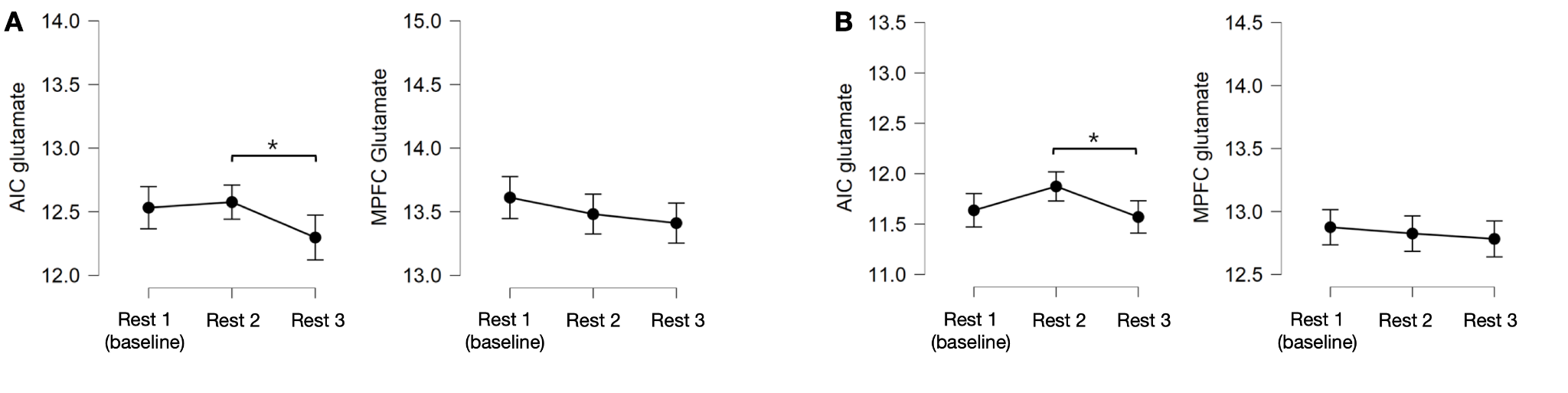
*

**Supplementary Figure 3.** Glutamate levels at three resting blocks (A: absolute, B: referenced to Cr+PCr). Error bars indicate confidence intervals of 95%. *p <.05


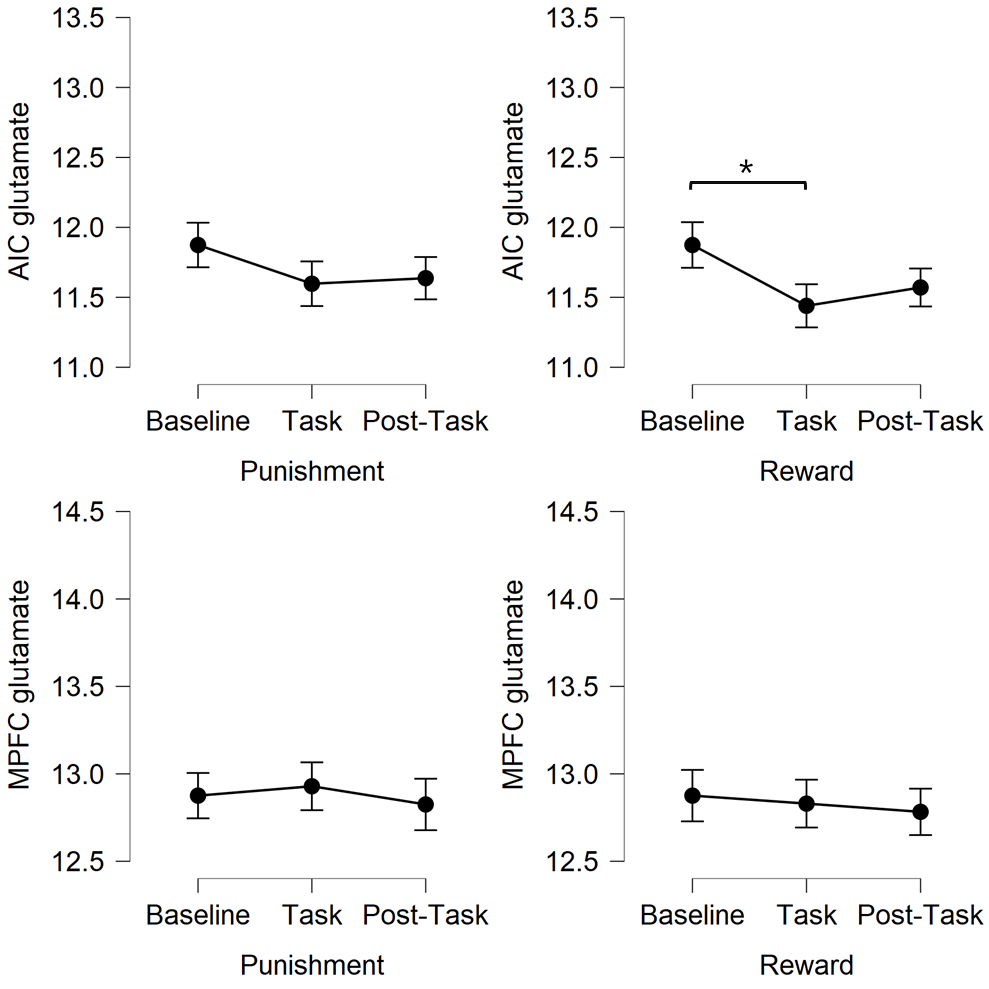


**Supplementary Figure 4.** Glutamate (referenced to Cr+PCr) concentration changes during learning


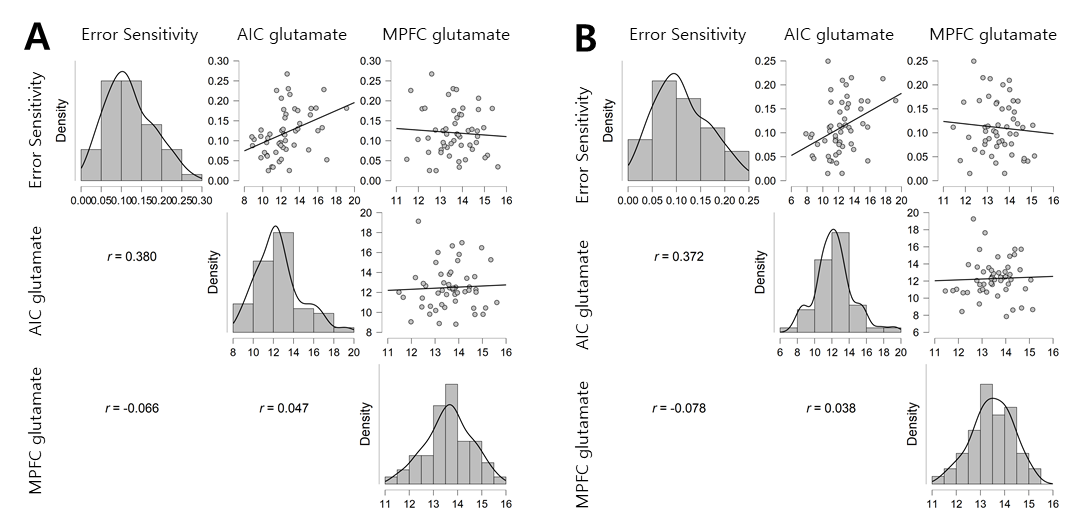


**Supplementary Figure 5.** Correlation plot for error sensitivity in (A) learning from losses and (B) learning from the gain with glutamate concentration in AIC and MPFC at the corresponding learning period.

**
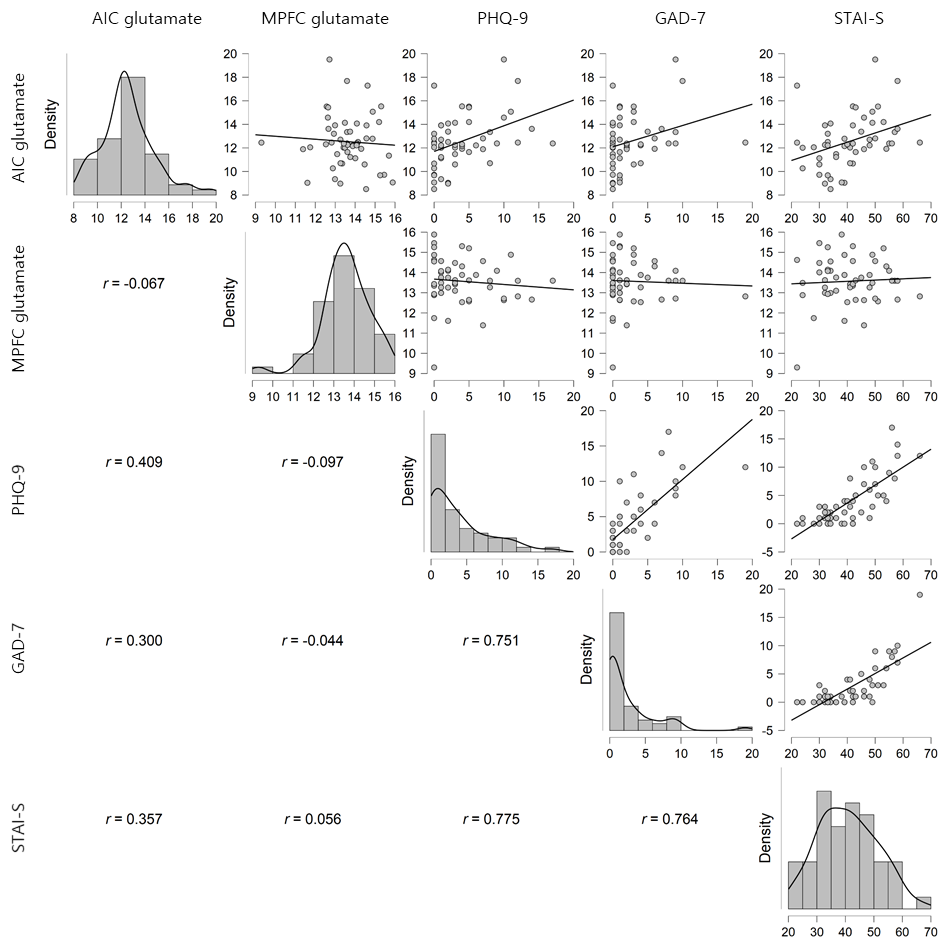
**

**Supplementary Figure 6.** Correlation plot for psychological state and glutamate in the AIC and MPFC.


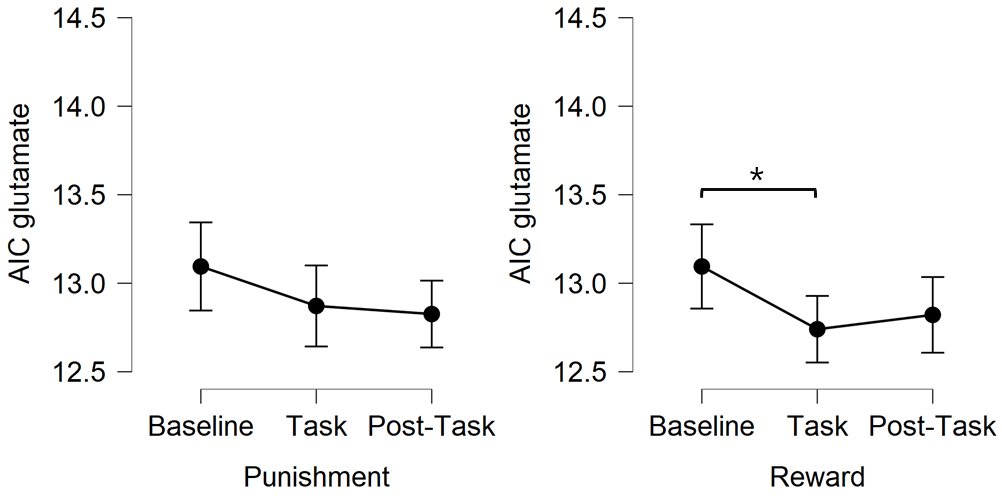


**Supplementary Figure 7.** Glutamate concentration in screened participants


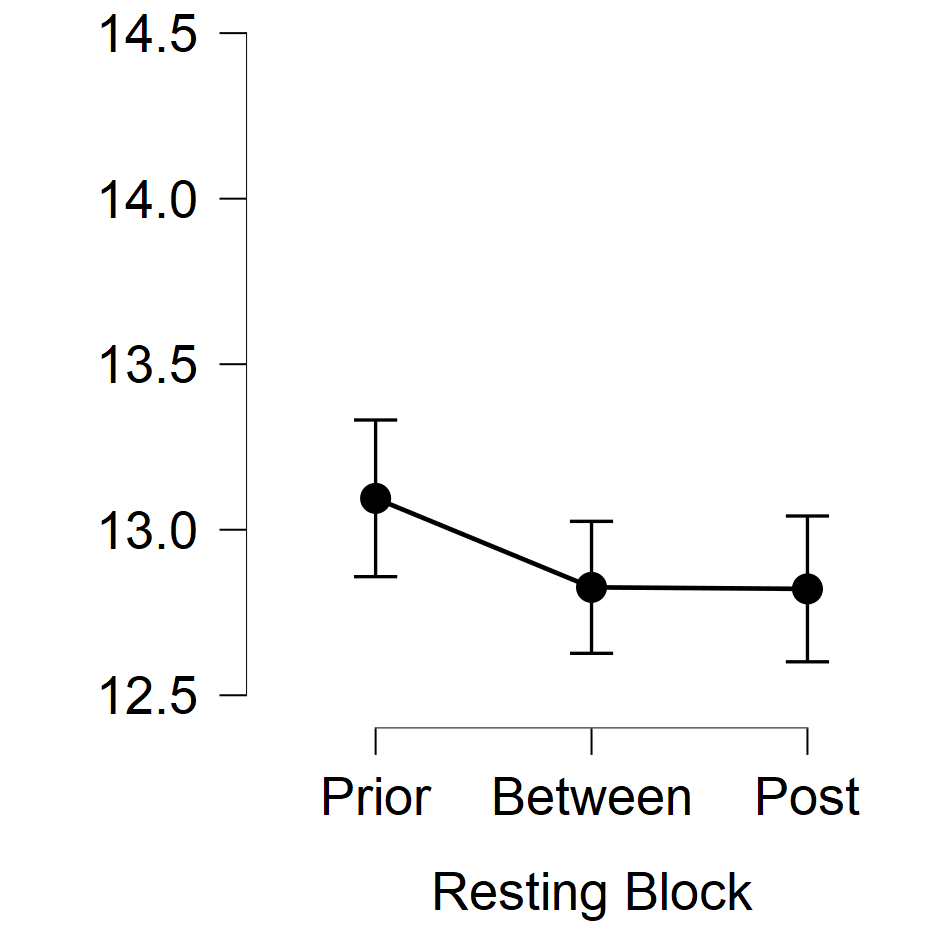


**Supplementary Figure 8.** Resting glutamate during resting.

Supplementary Figure 9. Correlation of AIC Glx and g-factor by error sensitivity group. Each group are dividied by median value of error sensitivity.


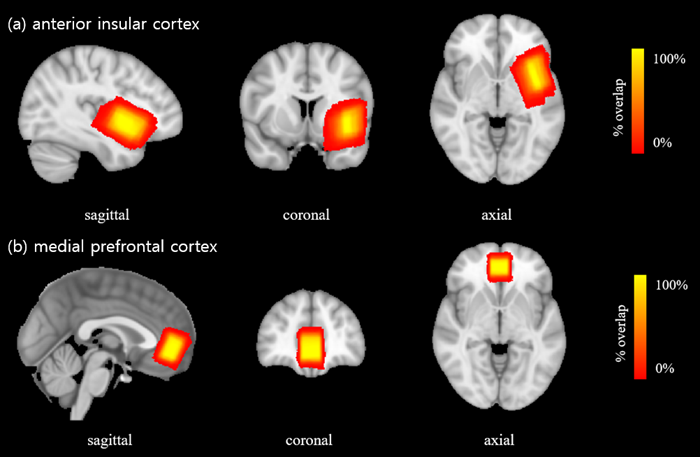


**Supplementary Figure 10.** Voxel placements of all subjects


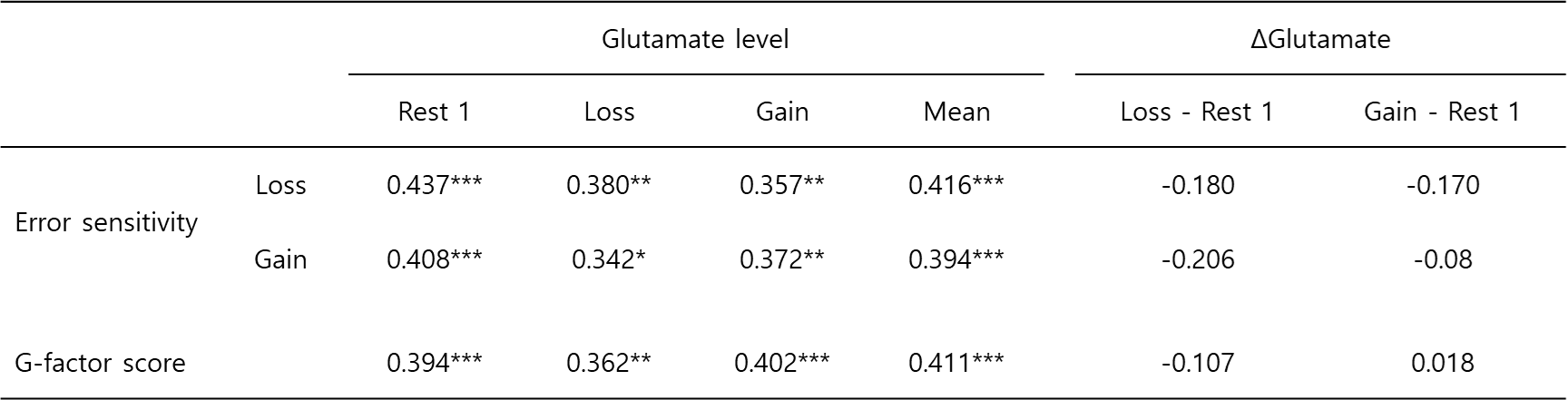


Supplementary Table 4. Pearson correlation between glutamate level or chanes in AIC and error sensitivity or g-factor score. 𝜟 Glutamate: calculated by subtracting the glutamate level of the Rest 1 (baseline) from the glutamate level of each task block. Mean: averaged glutamate level across the whole experiment consisting of all task and rest blocks. Regardless of the change according to learning, the higher the glutamate level in AIC, the higher the error sensitivity and g-factor score. Glutamate level or its change did not show a significant relationship with decision temperature or decay rate. *p<0.05, **p<0.01, ***p<0.005, Bonferroni corrected

Anderson, T. W., & Rubin, H. (1956). Statistical inference in factor analysis. *Proceedings of the Third Berkeley Symposium on Mathematical Statistics and Probability 111–150*.

Bornovalova, M. A., Choate, A. M., Fatimah, H., Petersen, K. J., & Wiernik, B. M. (2020). Appropriate use of bifactor analysis in psychopathology research: Appreciating benefits and limitations. Biological psychiatry, 88(1), 18-27.

Choi, K. S., Kim, S., Kim, B. H., Jeon, H. J., Kim, J. H., Jang, J. H., & Jeong, B. (2021, Aug 4). Deep graph neural network-based prediction of acute suicidal ideation in young adults. *Sci Rep, 11*(1), 15828. <https://doi.org/10.1038/s41598-021-95102-7>

Floyd, F. J., & Widaman, K. F. (1995, Sep). Factor analysis in the development and refinement of clinical assessment instruments. *Psychological Assessment, 7*(3), 286-299. <https://doi.org/Doi> 10.1037/1040-3590.7.3.286

Humphreys, L. G., & Montanelli Jr, R. G. . (1975). An investigation of the parallel analysis criterion for determining the number of common factors. *Multivariate Behavioral Research, 10(2), 193-205.*

Schmid, J., & Leiman, J. M. (1957). The development of hierarchical factor solutions. *Psychometrika, 22(1), 53-61.*
